# Informal ready-to-eat food vending: a social practice perspective on urban food provisioning in Nigeria

**DOI:** 10.1007/s12571-022-01257-0

**Published:** 2022-02-05

**Authors:** Kehinde Paul Adeosun, Mary Greene, Peter Oosterveer

**Affiliations:** 1 Environmental Policy Group, Wageningen University and Research Centre, Wageningen, The Netherlands; 2 Department of Agricultural Economics, University of Nigeria, Nsukka, Nigeria

**Keywords:** Out-of-home food provisioning, Ready-to-eat foods, Food diversity, Urban poor, Social practice theory, Nigeria

## Abstract

The way people access food in Nigeria is of central relevance for food security, health and sustainability. One key trend is the shift from household-based to primarily out-of-home food consumption as an increasing majority of the urban poor derive their daily nutrient intake from street foods. However, few studies have yet explored the role of the ready-to-eat food vending sector in urban food systems and the diets of the urban poor. This paper investigates the interrelations between these practices and the diversity of food groups provisioned among the urban poor in developing city contexts. A social practice approach is employed to explore differentiation among informal-ready-to-eat food vending practices in the city of Ibadan, Nigeria, in terms of their daily activities, competences and resources. Applied methods include GIS mapping, food log diaries, in-depth interviews and participant observation to map and classify informal-ready-to-eat food vending practices according to the nature of food provisioned and explore the everyday performances of different informal-ready-to-eat food vending practice initiatives and their relation to dietary diversity. The results reveal three key categories among these practices: traditional, processed and unprocessed—with varying levels of diversity in the food groups on offer. Traditional food vendors offer more diversified food compared to processed food vendors and unprocessed food vendors. The results reveal that material infrastructure, cooking bargaining and purchasing skills and nutritional knowledge are key to the diversity of food groups provisioned. The paper concludes by considering the wider relevance of these findings for urban food science and policy.

## Introduction

In urban settings in sub-Sahara Africa, in everyday life food is increasingly consumed out-of-home (Njaya, 2014; Mbah & Olabisi, 2015; Githiri et al., 2016; Hill et al., 2016; Swai, 2019; Tawodzera, 2019; Kolady et al., 2020; Wegerif, 2020). Recent studies (Esohe, 2012; Mbah & Olabisi, 2015; Ogundari et al., 2015; Resnick et al., 2019) have shown that nowadays informal ready-to-eat food vending practices (IRFV) constitute the most commonly used food provisioning system in in Nigeria, especially among the urban poor, who, due to hostile living and working conditions, are often unable to prepare their food at home. IRFV provide ready-to-eat foods and beverages sold on the streets and in small shops, motor parks, workplaces and even around schools (Muyanja et al., 2011; Hill et al., 2016). IRFV provides low-cost and convenient access to food, although often with a low diversity in terms of food groups, while allowing the vender to make a profit (Mwangi et al., 2002; Story et al., 2008). Informal ready-to-eat food vendors are not registered with either private organizations or the government (FAO-UN, 2016). Vendors don’t have any clear form of coordination and neither do they pay taxes or levies to the government. In Nigeria, about 51.7% of the urban out-of-home food consumers receive their entire daily food consumption from street food vendors (Mbah & Olabisi, 2015). Thus, for a large number of urban poor, the nutritional value and health of their diets depend on the food provisioned by informal food vendors (FAO-UN, 2015).

Accessing a diverse diet, necessary for a healthy life, is a serious challenge among the urban poor. This has raised concerns among policy makers and development practitioners, especially where food insecurity is prominent (Ahmed et al., 2015). IRFV practices have been identified as critical because of their increasing significance within urban food systems and the generally low diversity of the foods provisioned (Mwangi et al., 2002; Steyn et al., 2014). Research has shown that IRFV does not supply enough diverse foods and thereby contributes to poor diets among the urban poor (Mwangi et al., 2002; Pereira et al., 2005; Larson et al., 2009; Global Panel report, 2017; Tull, 2018). Informal food vendors bridge the gap of inadequate home food provisioning thereby meeting the food needs of out-of-home consumers. Thus, the nutritional health of out-of-home consumers depends on what food vendors are able to provision.

Due to their expansion and increasing significance, understanding IRFV practices is critical for informing urban food systems development. So far, most studies have taken an economic or nutritional perspective. From an economic perspective IRFV forms an important source of employment and income generation as well as a contribution to the livelihood of the poor (Chicho-Matenge & Nakisani, 2013; Charman et al., 2015; Petersen & Charman, 2018; Tawodzera, 2019; Resnick et al., 2019). From a nutritional perspective IRFV is studied to measure nutritional value and dietary intake among consumers (von Holy et al., 2006; Namugumya & Muyanja, 2011; Leshi & Leshi, 2017). Further work on informal markets in Nigeria has addressed food safety (Chukuezi, 2010; Dipeolu et al., 2007; Omemu & Aderoju, 2008) in connection with informal food retailing practices (Resnick et al., 2019). However, there has been a paucity of research exploring IRFV from a sociological perspective. As a result, little is known about the contexts in which everyday performances of IRFV are arranged and maintained and how this relates to its nutritional value. This study intends to address this gap. In doing so, it employs a situated social practice theory approach (Shove et al., 2012) to advance understanding the performance and context of IRFV practices and how they interrelate with the diversity of food groups provisioned within the broader frame of changing food environments. Our focus is on the role of the vendor as a critical actor in these practices.

A mixed methodology is employed to explore IRFV practices, including GIS mapping, food logging and qualitative inquiry. The case of the Nigerian city of Ibadan is taken because there is evidence that the low diversity of food groups provisioned by IRFV has contributed to weak dietary intake of poor people (Mbah & Olabisi, 2015). Nigeria is the most populated country in Africa and Ibadan has demographic and spatial arrangements comparable with most other cities on the African continent. Ibadan has the highest land mass among the cities in Nigeria where different sociocultural orientations are represented. Moreover, Ibadan is a prime example of a city with a complex infrastructure and high social dynamics providing the context in which IRFV takes place.

The paper continues after this introduction, with section two, where we introduce the social practice approach as our conceptual framework underpinning the investigation of IRFV. Section three then presents the multi-modal practice-centered methodology. Sections four and five present and discuss the results of this investigation and we conclude with reflecting on their relevance for future research and policy.

## Applying social practice theory to study informal food provisioning

This study investigates the operations and roles of IRFV in Ibadan. The overarching aim is to advance the debate on the capacity of ready-to-eat food vending-centered food systems to enable access to diverse diets by out-of-home consumers. As outlined, the paper takes a sociological practice-based approach to studying IRFV, drawing on Social Practice Theory (SPT), as a conceptual framework underpinning the investigation. SPT takes recognizable practices as the unit of analysis rather than individuals or social structures (Reckwitz, 2002; Wertheim-Heck & Raneri, 2019). By concentrating on the performative character of social life, SPT seeks to explore how agency and structural constituents of social life merge and interact in socially recognisable practices, understood as routinized ways of performing social activities, that persist and evolve over time. SPT offers a distinct approach to conceptualising social change, agency and action that departs from dominant reductionist or behaviourist accounts of action in social sciences. Instead of focusing on individual attitudes, beliefs, values or behaviours, practices are seen as the fundamental unit of the social world through which social change should be analysed. SPT allows us to understand and analyze food provisioning practices, as it makes up different practice-elements forming a “whole” practice and the integration of these practice-elements can be studied as competencies/skills, materials and meanings according to Shove et al. (2012, 2015). Furthermore, practice theory provides an attractive opportunity to study a practice beyond itself in connection with other practices as a bundle of practices. Even though a particular practice can stand on its own, however, sometimes, practice can connect with other practices for them to generate a consistent and complete meaning. Everyday actions are connected together in an array of practices, this means most times particular practices can be linked together to form a chain of practices that can be studied as bundled practices (Schatzki, 2014, 2016; Blue et al., 2016; Reckwitz, 2017).

SPT invites us to explore social life and change by examining how distinct practices are performed, maintained and changed over time, as well as how practices connect in a larger bundle of different practices. In recent years, SPT has been employed to analyze food production (food supply) and food consumption practices (Spaargaren et al., 2012; Welch & Warde, 2015, 2017; Shove, 2017; Welch & Yate, 2018; Wertheim-Heck & Raneri, 2019). Social practice allows us to understand the embeddedness of elements of practice:- competencies/skills, materials, and meanings within food provisioning practices. More so, Social practice provides a more comprehensive understanding of routine practices (as regards out-of-home food provisioning practices) and the integrations of different practice elements that form food provisioning. The study contributes to the body of literature as it holistically dissects food provisioning beyond the view of the combination of practice elements and goes further to examine the wider functions and performances of food provisions within the broader system of food provision. The study joins the body of literature that has employed social practice theory to analyze food provisioning. For instance, Cattivelli and Rusciano (2020) employed social practice to understand the configuration of social innovation in food provisioning. Gobbo et al. (2021) employed social practice to understand whether and how platforms make alternative “good food” more practicable. Likewise, social practice has been employed to understand the role of food safety in everyday food provisioning (Kendall et al., 2016). However, as of yet, little to no research has applied a social practice approach to understand IRFV practices.

At a very basic level, applying a SPT framework implies conceptualizing IRFV as a distinct social practice that is the outcome of daily performances and undergoes transformations over time. In studying IRFV as a social practice it is important to recognize that practices can be analyzed at various scales, from the analysis of elements of individual practices to explorations of interconnections and interlinkages between bundles of practices. For this end, social practices can be explored by switching between two analytical lenses, a zoomed-in and a zoomed-out perspective (Nicolini, 2012). Using a zoomed-in perspective, practices can be investigated by examining the integration of different practice-elements that make up a practice. Shove (2017) define the elements of a practice in terms of the constituent elements of meanings, materials and competences. Moving away from an analysis of specific practices, a zoomed-out perspective provides the opportunity to investigate a practice beyond its primary scope in its connection with other practices as part of broader bundles of practices. Alternating between a zoomed-in and a zoomed-out perspective enables the researcher to analyze the evolution of specific food provisioning practices, as well as their position within and interactions with, practice bundles constituting the dynamic interconnected social system (Nicolini, 2012; Wertheim-Heck, 2015).

**Fig. 1:**
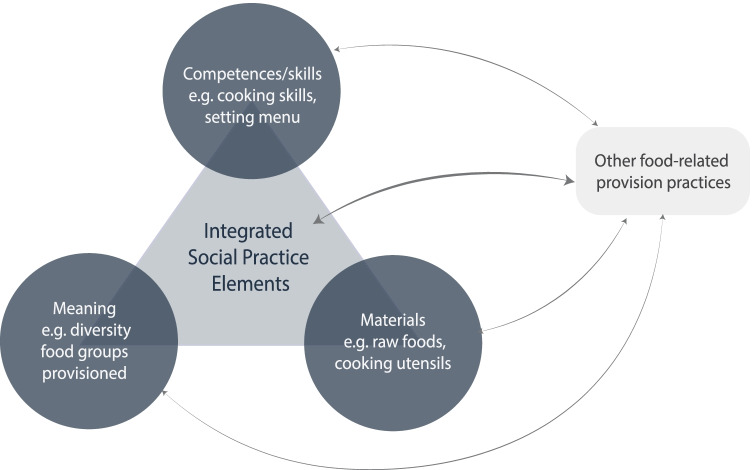
Interactions of practice-elements and arrangements bundles in informal ready-to-eat food vending practices

In this paper, ready-to-eat food vending is conceptualized as a social practice that integrates different practice-elements into an identifiable routinized activity (Shove et al., 2012, 2015). Building on Shove’s elemental model of practice-elements, this study analyzes ready-to-eat food vending practices as being composed of material objects and environments, as well as socio-cultural meanings and particular skills/competencies and capabilities (Shove et al., 2012; Walker, 2014). In performing IRFV, food vendors integrate and combine these various elements to produce variant food outcomes; that is, food products with different degrees of healthiness and diversity. This study seeks to analyse the dynamic interactions between the practice-elements of different vending practices to better understand the food diversity outcomes. Specifically, different food vending practices are explored in terms of the competences/skills/capabilities (i.e., cooking skills, nutritional knowledge, menu-settings, stocking/re-stocking, pattern and frequency of food provisioning, marketing skills etc.) employed to maintain the food vending practice, materials (i.e., procurement of food items, cooking utensils, storage and preparation space etc.) needed for their performance and the meanings attached to different activities and their influence (i.e. diversity of ready-to-eat food groups provisioned etc.) (Fig. 1) (Shove et al., 2012; Dobernig et al., 2016; Liu et al., 2016; Wertheim-Heck et al., 2019). Together these dynamic elements constitute a food vending practice and may produce varying capabilities to deliver diverse and healthy food products.

While this paper focuses on mapping the constituent practice-elements of different food vending practices, it also recognizes the embeddedness of the IRFV practices through their interconnections and interactions with other practices in the context of ready-to-eat food in urban Nigeria. The practice of ready-to-eat food vending takes place not in isolation but interconnects, interlinks, co-evolves and interacts with other (related) food provisioning and everyday life practices. In urban Nigeria, related food provisioning practices include wider agricultural and food supply practices involved in providing raw food ingredients through farming and wholesale markets. Other related practices interacting with IRFV include the wider social transformations that have provided the context for the emergence of IRFV. These include, rural–urban migration, changing urban lifestyles associated with working practices and transformations in daily urban commuting mobilities, and administrative and legal arrangements influencing food practices. These social developments interconnect and co-evolve with food vending practices and influence their functioning and performances in a dynamic way (Bhattacharyya, 2001; Dai et al., 2019; Swai, 2019; Zhong et al., 2019). For instance, rural–urban migration has been associated with significant transitions in food provisioning and consumption practices. Changing demand patterns associated with increases in populations of urban poor consumers have encouraged the expansion of food vending practices as more and more people demand their services (Swai, 2019). These developments have also been influenced by concurrent transformations in the economic organization of urban life; long commuting and working hours, as well as poor household and living conditions among the urban poor are key elements in the continued expansion of informal ready-to-eat food vending as urban life conditions force many people to eat out (Hill et al., 2016). Out-of-home food vending continues to prosper because many people use this as coping strategy within their urban lifestyles (Bhattacharyya, 2001; Ogundari et al., 2015). Changes in consumer demand associated with wider contextual developments interplay with food vending practices as food vendors try to adjust their practices to satisfy particular customer demands.

**Fig. 2:**
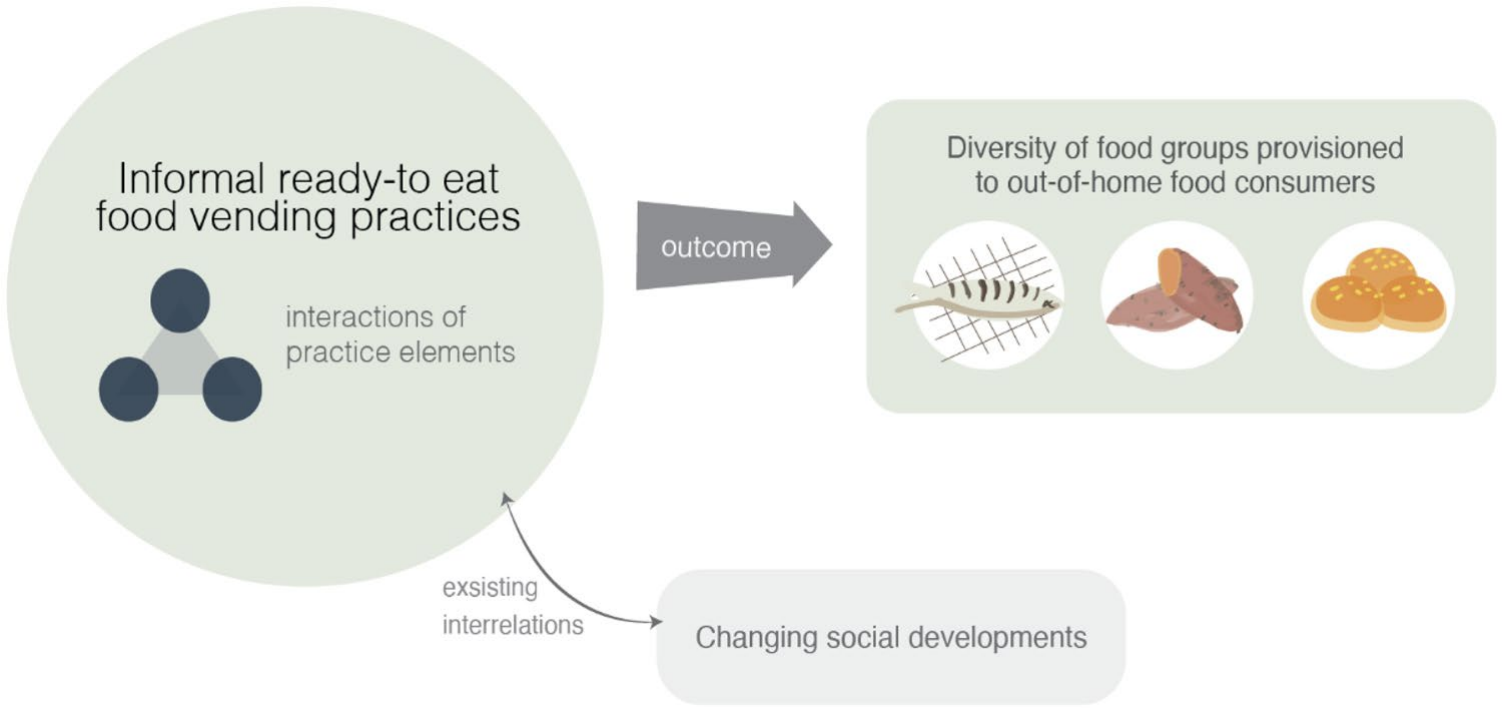
Dynamics interactions of food provisions practices within the food environment

Figure 2 presents the practice-based approach to studying IRFV in which different activities and practices of food vendors (for example, practices relating to food procurement, preparation, marketing, and food presentation) are explored for their influence on the diversity of the food groups provisioned. The number of food groups present in a diet, or in our case in the food items offered by a food vendor, is an indicator of the nutritional spread of a diet (Mwangi et al., 2002) and is measured by establishing the number of food groups present in a diet, or in our case in the food items offered by a food vendor. In general, 12 food groups are distinguished: (1) cereals; (2) tubers and roots; (3) legumes; (4) nuts, and seeds; (5) vegetables; (6) fruits; (7) meat; (8) eggs; (9) fish; (10) milk; (11) oils and fats; and (12) beverages (Sibhatu & Qaim, 2018; Rammohan et al., 2019). We focus in our research on the functions, performances and operations involved in IRFV practices and their relationship with the diversity of food groups provisioned, while recognizing the relationship between IRFV practices and wider changing social developments.

## Materials and methods

### Description of the study area

This research explores dynamics in ready-to-eat vending practices among low-income urban residents in Ibadan, a dynamic city with important social and economic functions. We selected the urban poor because they constitute the majority of the city inhabitants and are most vulnerable in terms of accessing a healthy and balanced diet. Even though some middle income groups also consume ready-to-eat foods, the majority of the out-of-home consumers belong to the low-income earners and the reason is that ready-to-eat food is cheap, so with little money they can buy food. This research builds on previous studies using nutrition and economic perspectives on food consumption in this urban area (Raaijmakers et al., 2018), in order to more explicitly advance insights into the social dynamics of food provisioning. Ibadan, a city experiencing rapid growth through a process of peri-urbanization, has a population of about 4 million (Adelekan, 2016). The city is highly multicultural, bringing together people from different cultures in Nigeria and around the world. Administratively, Ibadan consists of eleven Local Government Areas (LGAs), seven located in the Northern part of the city, three in the Southern part and one at the boundary between the Northern and Southern parts.

### Research participants, sampling and data collection strategies

The informal food vendors included in this study are individuals directly in control of food vending outlets and in operation for at least the past five years. They operate in low-income communities in Ibadan, communities where most people live below the urban poverty line of $1.90 per day (UNDP, 2004). The sample for this study was selected on the basis of information from the state ministry allowing us to categorize the Local Government Areas (LGAs) in Ibadan. Based on this information, we selected the lowest income LGAs for further investigation. A preliminary survey was conducted during which community leaders were consulted. They were provided with an information sheet detailing the purpose and expected outcomes of the research, and how their involvement would facilitate its success. To have a good representation of the study area, we selected two LGAs from the Northern part of Ibadan where seven LGAs are located, one LGA from the Southern part of Ibadan where three LGAs are located and one LGA from Central Ibadan, located at the boundary between the Northern and Southern parts of the city. Thus, in total, four LGAs were selected as the sample area for the study and within each of them, two poor urban communities were selected as sites for the study (See Table 1).

**Table 1:** Showing the selected LGAs and communities in the study area

**LGAs**	Ibadan north west (central)	Lagelu (north 1)	Ido (north 2)	South west
**Communities**	Ologuneru	Akobo	Apete	Odo ona
	Eleyele	Iyana church	Apata	Osasimi

**Fig. 3:**
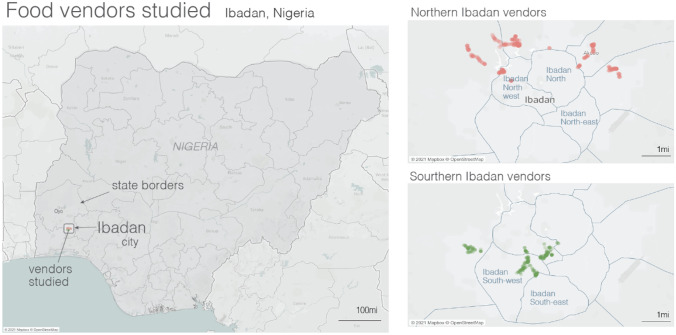
GIS mapping of food vending outlet’s locations in Ibadan

Geographic information System (GIS) was applied to map the location of IRFV-outlets in these communities and establish their spatial distribution in the study area (See Fig. 3). A research team of four trained assistants moved around to identify and take the coordinates of all IRFV-outlets in the selected communities. Out of about 686 food vending outlets mapped in the selected communities, the study selected 100 food vendors for the qualitative research and food logging activities.

**Fig. 4:**
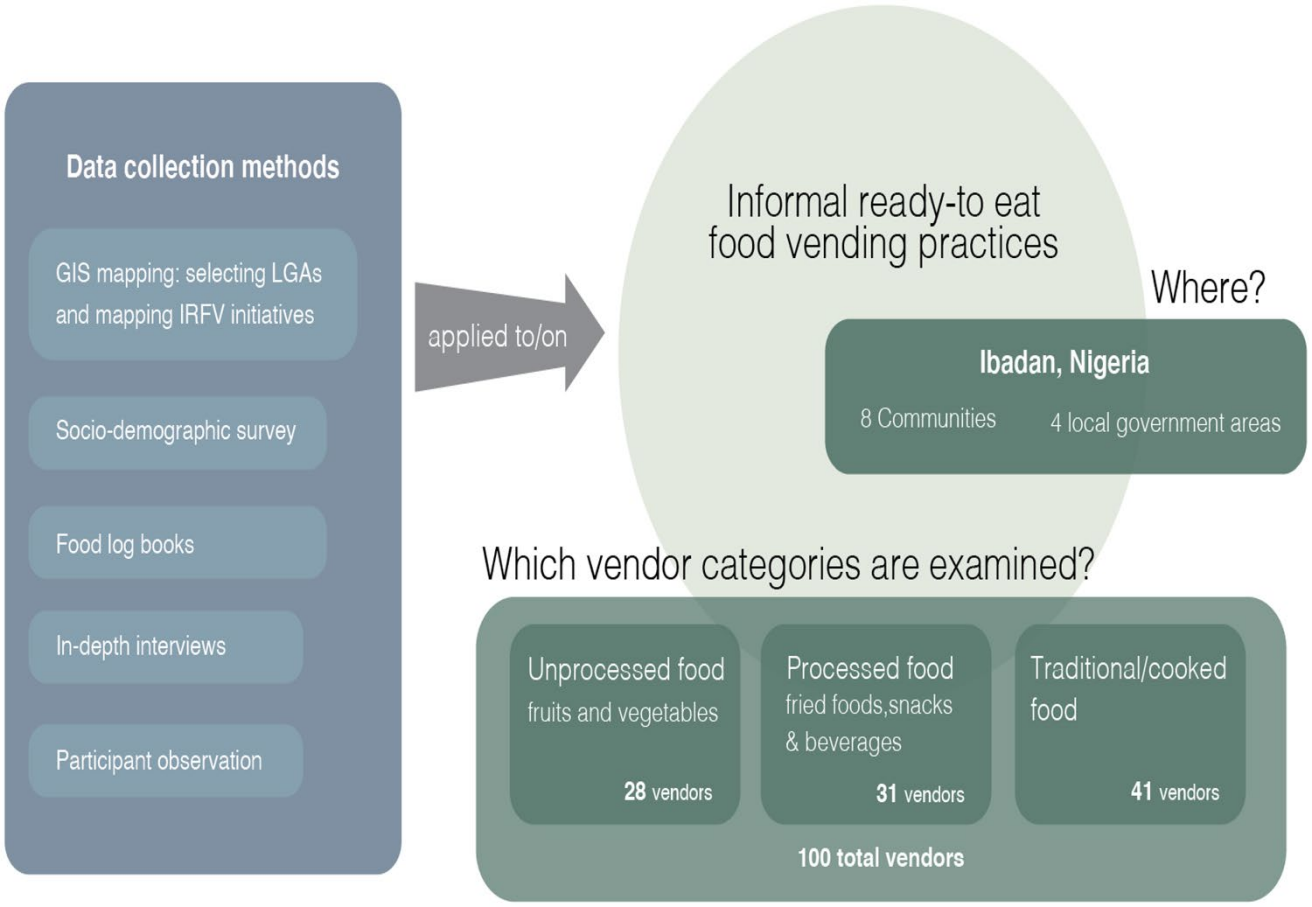
Sampling procedure

Following the GIS mapping, we deployed a stratified sampling strategy to select our respondents, based on the categorization of IRFV using the kinds/forms of foods provisioned (Steyn et al., 2014). The three identified categories are: (1) traditional cooked meals (local foods such as cooked rice, yam, soup, pounded yam, eba and amala; (2) processed foods (fried foods, snacks and beverages); and (3) unprocessed foods (fruits and vegetables) (Steyn et al., 2014). In this study, traditional food is refer to as locally made meal, while processed food is mostly snacks and highly processed and fried food. Linkages with food vendors were established mainly through community leaders who are well known in the communities and hold strategic positions. During this process, we generated a list of all informal ready-to-eat food vending outlets that formed our sample population. The selection criteria included vending category type (we sought a more or less balanced representation across the three categories) (See Fig. 4) and the readiness of the informal food vendor to participate in the study. 100 respondents across the selected communities from the inventory generated comprised our sample and were invited to participate. The selection was done based on the proportion of food vending categorizations in the selected communities. We found traditional food vending to be the highest number followed by processed and unprocessed food vending and on this basis we selected our respondents. Verbal and written consent was obtained from all respondents prior to their involvement in the study and respondents were informed that they could discontinue participation at any stage if they wished to.

The research team, composed of four well-trained research officers and the principal researcher, collected data in the period from August to October, 2020. Although this study was conducted during the COVID-19 pandemic, at the time of the investigation all lockdown measures had been lifted and food vendors were officially allowed to operate. In addition, all COVID-19 precautionary measures were undertaken, such as keeping 1.5 m social distance and wearing of face masks by the respondents and the interviewers.

Primary data collection included semi-structured interviews, food logs and passive participant observation. (See Fig. 4) All methods were implemented concurrently and provided complementary insights into the everyday practices of IRFV practitioners. Interviews with respondents were structured to generate insights into informal ready-to-eat food vending while also observing their activities and operations during the interview. Respondents were questioned on their operations, activities, and functions to understand how these related to the diversity of food groups provisioned. Each interview lasted approximately 30 to 45 min and was performed at respondents’ vending location. Interviews were audio recorded for transcription purposes. Data on situational dynamics that was difficult to collect through interviews was generated through food logs and observations. Data from participant observations was recorded in field notes during and immediately after the interview. The interviews and participant observation data were transcribed and coded using the qualitative data analysis software program Atlas.ti. The analysis followed iterative movements between coding inductively for particular themes and patterns and then reviewing and further categorizing this data according to the social practice theory conceptual framework.

### Measuring diversity of ready-to-eat food groups provisioned

In this study, we focused on the food diversity of street food providers rather than the consumers because they determine the composition of the menus available to out-of-home consumers. This means that the choice of a variety of foods on their menu list is their sole decision and consumers can only choose from what is available. Street food is very important in the daily diets of Ibadan citizens because this is where the majority of out-of-home consumers arrange their major nutrient intake. As most of the out-of-home consumers have a particular food vendor they eat from, it is important to analyze food groups' diversity at the level of the individual vendor.

Consequently, the details of the diversity of the food groups provisioned and menu settings/stockings were obtained using food-type-log-book within seven (7) days of practice. The measurement of food diversity was based on the 12 different food groups mentioned above and these were analyzed based on the groupings of ready-to-to-eat food vending. The Dietary Diversity Score (DDS) measurement was adapted to measure the diversity of food groups provided by food vendors based on “count”. The diversity of food groups provisioning was descriptively analyzed based on four discrete categories: lowest diversity = 5 or below; Mid Low diversity = 6; Mid High diversity = 7; and Highest diversity = 8 and above (Pritchard et al., 2019; Rammohan et al., 2019). The indices measure the overview of the diversity of food groups provisioning by the food vendors. Although DDS has been commonly used to measure the diversity of diets using a consumption perspective, this study adapted this tool to measure the diversity of ready-to-eat food groups from a provisioning perspective.

## Results

The results of the investigation are presented in two steps. First, an overview is provided of the IRFV initiatives in terms of their categorization based on the degree of diversity of the food groups provisioned. Second, we move to more explicitly unpacking and exploring these categories of IRFV practices according to their constituent practice elements. Drawing on the interview data and participant observations, an analysis is presented of the contexts and performances of IRFV practices and their relation to dietary diversity outcomes.

### Profile of informal food vendors

Table 2 summarizes the key socio-demographic characteristics of the vendors included. Our sample shows some points of homogeneity, such as age and gender. The majority of people involved in the practice of IRFV are between 34 – 45 years old. This corresponds with previous findings suggesting the majority of IFV practitioners are young adults (Dipeolu et al., 2007). Furthermore, the vast majority of food vendors are women, ranging from 83.9% to 96.4%. Also a majority of the food vendors (85.4% of TFVs, 74.2% of PFVs and 78.6% of UPFVs) listed ready-to-eat food vending as their primary occupation and source of income. Furthermore, most food vendors sell their food from a fixed stationary location, mostly along the streets, with some conducting their business through (semi-)mobile operations, confirming the findings of Simopoulos and Bhat (2000).

**Table 2:** Socio-economic characteristics of informal food vendors

**Food vendor’s characteristic**	**freq**	**%**		**freq**	**%**		**freq**	**%**
	**Traditional vendors**			**Processed food vendors**			**Unprocessed food vendors**	
**Age**								
21—33	3	7.3		3	9.7		3	10.7
34—45	18	43.9		14	45.2		12	42.9
46—58	10	24.4		10	32.3		8	28.6
59 and above	10	24.4		4	12.9		5	17.5
**Workers**								
< = 1	15	36.6		19	61.3		19	67.9
2—4	22	53.7		6	19.4		8	28.6
5—8	2	4.9		3	9.7		1	3.6
9 and above	2	4.9		3	9.7		-	-
**Food vending experiences**								
= 5	3	7.3		7	22.6		6	21.4
6—17	18	43.5		17	54.8		13	46.4
18—28	14	34.6		5	16.1		5	17.9
29 and above	6	14.6		2	6.5		4	14.3
**Sex**								
Female	39	95.1		26	83.9		27	96.4
Male	2	4.9		5	16.1		1	3.6
**Marital status**								
Single	-	-		2	6.5		2	7.1
Married	36	87.8		25	80.6		23	82.1
Widow	5	12.2		4	12.9		3	10.7
**Occupation**								
Primary	35	85.4		23	74.2		22	78.6
secondary	6	14.6		8	25.8		6	21.4
**Education**								
No formal education	1	2.4		3	9.7		6	21.4
Primary	6	14.6		7	22.6		9	32.1
Secondary	26	63.5		9	29.0		10	35.7
Tertiary	8	19.5		12	38.7		3	10.7
**Type of food vendor**								
Mobile	-	-		2	6.5		-	-
Stationary	36	87.8		26	83.9		20	71.4
Semi-mobile	5	12.2		3	9.7		8	28.6
**Period of provisioning**								
Morning	5	12.2		2	6.5		-	-
Whole day	24	58.5		15	48.4		25	89.3
Morning and afternoon	9	22.0		8	25.8		-	-
Afternoon and evening	3	7.3		4	12.9		3	10.7
Morning and evening	-	-		2	6.5		-	-
**Location**								
Along the road	11	26.8		15	48.4		19	67.9
Market square	4	9.8		1	3.2		1	3.6
Along the street	25	61.0		13	41.9		8	28.6
Motor park	1	2.4		1	3.2		-	-
Work place	-	-		1	3.2		-	-
**Total**	**41**	**100**		**31**	**100**		**28**	**100**

### Diversity and health of food groups provisioned

In order to map the dietary diversity outcomes across the three key categories of IRFV practices, that is traditional, processed and unprocessed food vendors, as an indicator of the health of their food on offer, we established the number of different food groups provisioned by the vendors. The food-type-log-books maintained over a period of seven days of practice, generated data on the diversity of the food groups.

### Diversity classification of food groups provisioned by food vending practices

The Dietary Diversity Score (DDS) is commonly used to measure the diversity of diets from a consumer perspective but in this study we adapted this tool to measure the diversity of ready-to-eat food groups from a provider perspective. Considering the high dependence of out-of-home consumers on informal food vendors for supplying their dietary needs, the number of food groups forms a useful indicator of their average dietary intake. The DDS was calculated by counting the number of food groups provided by the food vendors.

Table 3 presents the DDS for the different categories of food vendors. Our findings indicate that the different categories of IRFV provide diverse food groups in varying numbers and mixtures. The table shows that 31.71% of the (41) traditional IRFV provisioned seven food groups compared with only 9.68% of the (31) processed IRFV. The largest group of the processed IRFVs (29.04%) provisioned five food groups, whereas the largest share of the (28) unprocessed IRFVs (46.43%) provisioned only one food group. Overall, 77.42% of the processed and all unprocessed IRFVs were classified with low DDS (below 5 food groups). In contrast, only 7.32% of the traditional IRFVs scored low while 78.05% scored high DDS (7 or more food groups). These findings indicate that, in general, traditional IRFVs provisioned the largest number of food groups by far and thus provide meals with the largest food diversity.

**Table 3:** Diversity of food groups provisioned by ready-to-eat food vendors

**Traditional meal vendor**			**Processed food vendor**			**Unprocessed food vendor**		
**Food groups provisioned (count)**	**freq**	**% of traditional meal vendor**	**Food groups provisioned** **(count)**	**freq**	**% of processed food vendor**	**Food groups provisioned** **(count)**	**freq**	**% of unprocessed food vendor**
5	3	7.32	1	1	3.23	1	13	46.43
6	6	14.63	2	2	6.45	2	10	35.71
7	13	31.71	3	6	19.35	3	5	17.86
8	7	17.07	4	6	19.35			
9	4	9.76	5	9	29.04			
10	5	12.20	6	2	6.45			
11	3	7.32	7	3	9.68			
			8	2	6.45			
**Total**	**41**	**100**		**31**	**100**		**28**	**100**

Explanation: This analysis is based on the 12 food groups identified in the literature (Sibhatu & Qaim, 2018; Rammohan et al., 2019): cereals, roots and tubers, beans and legumes, nuts and seeds, fruits, meat, fish, oil and fats, milk, beverages, eggs, vegetables.

### Health classification of food groups provisioned by food vending practices

Besides their diversity, the food groups were also classified into healthy, neutral and unhealthy. Following the classification of the High Level of Expert Panel on food and nutrition (HLPE, 2017), vendors were scored based on the nature and types of food items provisioned from each food group. From the twelve food groups in total, seven are considered healthy, three neutral and two unhealthy (See Table 4).

**Table 4:** Showing classifications of food groups provisioned based on healthy, neutral and unhealthy foods

**Healthy food groups**	**Neutral food groups**	**Unhealthy food groups**
beans and legumes	cereals	meat
fish	tubers and roots	oil and fats
eggs	beverages	
fruits		
vegetables		
milk		
nuts and seeds		

Table 5 presents the food vendors in the different categories providing the different healthy, neutral and unhealthy food groups. Traditional IRFV provision predominantly beans and legumes, fish and eggs as health food groups, cereals and tubers and roots as neutral foods, and meat and oil and fats as unhealthy foods. Processed IRFVs provisioned predominantly fish and eggs as healthy foods, cereals as neutral food and oil and fats as unhealthy food groups. Unprocessed IRFVs provisioned predominantly fruits and vegetables, and also nuts, as healthy foods, and no neutral or unhealthy foods. Among the traditional IRFVs the highest number of food vendors provisioning healthy food groups can be found.

**Table 5:** showing the percentage of food vendors provisioning healthy, neutral and unhealthy foods

Healthy foods															Neutral foods						Unhealthy foods			
	Beans and legumes		Fish		Eggs		fruits		Vegetables		Milk		Nut and seeds		cereals		Tubers and roots		beverages		meat		Oil and fats	
**Category of food vendors**	**Freq**	**%**	**Freq**	**%**	**Freq**	**%**	**Freq**	**%**	**Freq**	**%**	**Freq**	**%**	**Freq**	**%**	**Freq**	**%**	**Freq**	**%**	**Freq**	**%**	**Freq**	**%**	**Freq**	**%**
**TFV (n = 41)**	**34**	**82.9**	**34**	**82.9**	**28**	**68.3**	**21**	**51.2**	**24**	**58.6**	**Nil**	**Nil**	**1**	**2.4**	**36**	**87.9**	**32**	**78**	**6**	**14.6**	**41**	**100**	**41**	**100**
**PFV (n = 31)**	**9**	**29**	**17**	**54.8**	**19**	**61.3**	**6**	**19.4**	**3**	**7.7**	**6**	**19.4**	**9**	**29**	**26**	**83.8**	**7**	**22.6**	**5**	**16.1**	**7**	**22.6**	**26**	**83.9**
**UPFV(n = 28)**							**28**	**100**	**28**	**100**			**12**	**42.9**										

### Exploring dynamic elements of food provisioning practices

Drawing primarily on qualitative interviews and participant observations, this section examines the context, performance and situated dynamics of different IRFV practices in Ibadan.

#### Relevance and informality of ready-to-eat food vending practices

Overall the study confirms previous findings on the centrality and relevance of street food (Swai, 2019; Tawodzera, 2019) in the daily diet of Ibadan citizens, particularly among the urban poor. Food vendors indicate that they have a consistent and regular consumer base and suggest that, for many of their clients, this is where they derive the majority of their daily nutrient intake. Most of the out-of-home consumers have a particular food vendor from whom they regularly buy their food, confirming the importance of analyzing food group diversity at the level of food vendors.

Informality in ready-to-eat food vending cuts across the different elements of food vending activities, from arrangements with customers, to choices concerning the kind of food they provide, to wider legislative contexts and relationships with public authorities. No formal regulations guide the interactions and interrelations between food vendors and their customers nor the food provisioned. Informal food vendors do not register with the authorities and do not pay taxes, so they do not have to follow a set of formal legislative or administrative rules guiding their operations.

The majority of the food vendors regard their food vending operation as their main job and chief source of income. This influenced their commitment and involvement in food vending as well as the level of competence and skill they feel they embody.

*“This is the job I have been doing from the beginning of my life and because of my long experience, I know the kinds of foods my customers want, thus I cook mainly what interests my customers (TFV 1, age 48)”.*

For traditional food vendors in particular, increased experience leads them to provision more common food items, although not necessarily more diverse as the food items provisioned may still be from a particular food group. For instance, a traditional IRFV may cook different products on the basis of cassava which does not mean more dietary diversity. However, these are common foods that people prefer to eat in that food environment. In this respect, experience gained from continuous practice, as well as interacting with consumers requesting additional food products, were reported as key factors influencing the expansion of the types of food provisioned.

The vending activities are embedded in the wider family lives of the food vendors; most of the respondents received support from members of their family (Simopoulos & Bhat, 2000). On the other hand, families also profit as was shown among traditional food vendors where the majority of their children received their daily meal from what was provisioned at the vending operation. This may likely influence the diversity of food groups provisioned; some vendors also aim to supply the required nutrient intake of their children directly or indirectly through the food they provide and, for some, this increases the likelihood that they are concerned with improving the diversity of the food groups provisioned. However, most traditional IRFV provisioned the kinds of foods their customers prefer, so their families also has to eat these.

#### Application and transmissions of skills and competences

Key differences between the categories of IRFVs exist in terms of the types of skills and competences needed to sustain their operations. Traditional food vending requires more skills and competences when compared with processed and unprocessed food vending. Traditional food vending includes more food groups in their menus allowing customers more choice. This difference in skills and competences has implications for the differences between IRFV types in terms of pathways of recruitment, training and learning.

Most traditional IRFV reported being recruited into the practice through informal learning processes within the family. Many had a mother, aunt or sister already engaged in the practice and, through observing, social learning and participating whenever the practice was being performed, they became increasingly acquainted with the practice. Most traditional IRFV respondents view the transmission of food vending skills and competences as guided by generational connections, in the sense that the practice is being transferred from one generation to another within the (nuclear or extended) family.

*“…I was informed by the practice from my mother as I usually participated in the activities. I usually observed my mother doing the activities (TFV 5, age 40).”*

Still, some traditional food vendors acquired the necessary skills through apprentice by being a support worker. When they have learned the necessary skills and competencies, they start their own independent IRFV themselves.

In the case of processed IRFV, socialization and recruitment through family ties was less common and instead many entered the practice based on what they had learned about food preparation in school. In comparison with traditional IRFV, unprocessed IRFV requires less competences and transfer of skills, thus enabling easier and faster recruitment. Unprocessed IRFV essentially required a brief consultation with existing practitioners particularly on procurements and pricing skills. They enter the practices without much specific training.

All three categories of food vending require skills such as purchasing and bargaining, customer treatment/relationship, nutritional knowledge, marketing, and being proactive. Cooking skills are common for traditional IRFV, while, measuring and baking skills are more common for processed IRFV and ability to detect spoilage for unprocessed IRFV. Our findings show that nutritional knowledge, purchasing and bargaining skills and cooking skills influence the variety of food groups provisioned. Overall, the more diversified the food vending practice, and the more complex the skills and competences underpinning it, the higher the earnings the vendor receives.

#### Food provisioning period/time

Timing in out-of-home food provisioning practices refers to the time of the day at which different food items and meals are provisioned. Some food items are provisioned in the morning because they are culturally considered breakfast, whereas some other food items are considered evening food. Traditional IRFV practices include providing rice, beans, spaghetti and stew in the morning and mashed foods, such as pounded yam and processed cassava, in the afternoon and evening because they are seen as heavy foods with high energy content. Processed and unprocessed foods are in demand throughout the day and not linked to particular meal times as they provide fewer food items. Most food vendors provision foods from Monday to Saturday and take Sunday off because they go to church. After church service on Sunday only a few food vendors are present, mostly selling processed and unprocessed foods, such as fried foods, noodles and fruits. Overall, we found that traditional food vendors, providing the most diverse foods, follow culturally and institutionally conditioned food timing more strictly. Furthermore, the timing also influences the diversity of the food items provisioned. For instance, in the case of traditional IRFVs fewer food items are usually available in the morning compared to the afternoon and evening when people have more choice.

#### Material resources: Procurement of raw materials

Sourcing material inputs is an important component of food vending practices that interrelates with the kinds of foods groups provisioned. Different categories of food vendors source their inputs from different markets depending on the food groups on offer. For instance, traditional IRFVs purchase their inputs at open markets where raw local food materials are sold. In contrast, processed IRFVs are more likely to buy their food materials from grocery shops. Unprocessed IRFVs mostly buy directly from farmers at the farm gate because this is cheaper. Generally, raw food materials are sourced on a weekly basis or when stocks are nearly finished. However, a common practice among traditional and processed IRFVs is to purchase perishable food products such as vegetables every day because they spoil very quickly. Sometimes, particularly traditional IRFVs make logistical arrangements with their suppliers to deliver produce which reduces the stress of having to go to the market every time to buy food inputs and also provides more time for vendors to concentrate on food preparation and selling activities.

*“…I have suppliers for all my raw materials. All I have to do is call the different suppliers of the raw materials on the phone once I am running out of stock and it is brought directly to me (PFV 8, age 35).”*

On the other hand, other respondents preferred to adopt a more flexible approach to choosing a supplier, depending on the conditions of the market:

*“... I do not really have a particular supplier I patronize in the market because when the price of things increase they are quick to tell you, but when there is reduction in the price, they will not tell you and still sell it at a higher price,” (TFV 7, age 42).*

Adopting a flexible approach is a common practices among both traditional and processed IRFVs because food preparation competes for time with food procurement, leading some vendors to opt for deliveries.

The availability of different raw food materials within the systems of provision influences the practices of food vendors, including the combination of types of food provisioned. At times of high demand, food vendors might buy inputs from grocery shops and small markets close to them, particularly when they need to buy in small quantities. However, if inputs are unavailable this may result in reduced diversity of foods available at a particular vendor until the food group can be sourced again.

#### Informal ready-to-eat food vending interrelates with other food-related provisioning practices

Our findings indicate that a dynamic interconnection exists between IRFV practices and other food-related provisioning practices. Food vendors source parts of their food ingredients from other food outlets and the vendors who the IRFV's buy ingredients from in turn buy ready to eat foods from them.

Together they operate a kind of vendor-vendor relationship.

*“…Having them around particularly the food groceries shops help me to quickly get what I want instead of working or travelling a far distance to market (PFV 15, age 37).”*

There are some supermarkets and restaurants around the IRFV locations, but consumers do not visit them because they are quite expensive. Nevertheless, IRFVs imitate them or are inspired to innovate in order to stand out:

*“...when I started at this location, I decided to be innovative and do things differently because of the environment i.e. the hotel and beer parlour around me. I started adding vegetables like cabbage, runner beans, carrot, cucumber, onions etc. I even started frying chicken to sell. I garnish my noodles with vegetables, and crayfish (PFV 16, age 45).”*

As this processed food vendor’s account suggest, competition in the wider food environment can act as a driver for increasing diversity of the food groups provisioned. The respondents agreed that good relationships exist between IRFVs and raw food materials vendors. At times, they give them inputs on credit and will be paid back later. However, most of the time unprocessed IRFVs buy their fruits in bulk directly from open markets or at the farm gate. Having other food outlets around provides quick access to food raw materials and also enables provisioning a larger variety of food groups.

#### Stocking, re-stocking and storage practices of ready-to-food vending

The practice of stocking involves keeping raw food materials in the shop or in a safe place to allow for preparing small quantities. Most food vendors indicated that they stock their raw food materials to prevent having to go to the market on a daily basis. They take the quantity they need to prepare for the day until their stock is almost finished. However, some food items cannot be stocked for more than a day or two at the most before being spoilt. Unprocessed IRFV cannot stock their fruits for too long because otherwise it will ripen and spoil. However, they preserve their fruits under shields and in cool places to prevent spoilage. Both the traditional and processed IRFVs report that, apart from particular food items that need to be sourced every few days, on average they stock their food ingredients for about 5 days to one week before needing restocking. The food materials are stocked in the shops in a cool environment.

*“...I restock when the available ones have almost finished. I go to the market every 5 days. I do not allow the available food materials to finish before I buy another, when gauge with my eyes and realized the remaining will only last a day or two, I must buy another one (TFV 21, age 52).”*

Stocking influences the provisioned diversity in food groups as it encourages food vendors to buy a greater variety of food items that can be stocked for days without spoiling. Thus, a greater capacity to stock and store food raw materials appears to increase the variety of ready-to-eat food groups provisioned.

The storage practices of IRFVs also include preservation of already prepared ready-to-eat food items and meals. In general, meals need preserving to allow selling the next day and this depends upon the availability and access to equipment such as cooling boxes, fridges and freezers. These storage practices varied among vendors depending on the types and diversity of the food groups provisioned. For traditional IRFVs, food items like semovita, pounded yam and beans cannot be sold the following day, whereas food items like rice and fufu can be stored and sold the following day. For processed IRFVs, snacks and fried foods generally cannot be stored and preserved and must be consumed on the day of preparation, otherwise the customer will detect the decline in quality and taste. The majority of the traditional IRFVs do not store their foods at all but ensure they produce the food products they can and sell in a day to avoid the need for storing until the following day. However, some traditional IRFVs did engage in storage practices by using freezers to store surpluses of prepared foods.

The majority of the food vendors reported that if there are leftover foods, they either give it away or serve it to their family. Unprocessed IRFV are not concerned about leftovers, since unsold fruits can be stored easily in boxes in a cool environment. However, if the fruit stays too long, the unsold items may perish or over-ripen and can no longer be sold. Thus, storing practices can promote the diversity of food group provisioned in the sense that, when food vendors are assured that if their foods do not finish and can be stored overnight, they can provision more food groups.

#### Food preparation

Food preparation, an important component of food vending practices, differed between the food vending categories and according to the type and diversity of the foods being provisioned. We found differences in the preparatory procedures and processes, including the utensils used and the raw food materials needed. Both traditional and processed IRFV begin with preparing food in the morning, as early as six o’clock, whereas unprocessed IRFV does not need any preparation other than putting the fruits on the tray. In the case of traditional IRFV, certain preparation stages are being done the previous day to ensure that the food is ready as early as possible to supply for breakfast customers:

*“I do some of my food preparation the previous night, such as frying of meat, grinding of pepper, etc. To make things easier, I always boil my pepper at night (TFV 13, age 38).”*

Food preparation continues in the food vending outlets throughout the day as vendors sell food. Food items are prepared in a continuous manner in response to demand, cooked bit-by- bit and as a particular food item is about to finish another batch is put on the fire. Likewise, food items are left on a mild fire to keep them warm and ready for sale.

*“…I cook the foods a little at a time, I start the preparation of another batch of food item when I perceive is about to finish (TFV 26, age 50).”*

According to the respondents, preparing some food items such as pounded yam and fufu is labor-intensive, so they are often excluded from the menu or the preparation is outsourced. Compared with processed and unprocessed vendors who report spending less time in preparing a smaller range of food groups, it is more rigorous and time consuming for traditional IRFV to prepare their food because of the number and complexity of steps involved in preparing more complete dishes from a larger number of food groups.

#### Menu-settings

Menu setting is the presentation of the food provisioned by the vendors. Each category of food vending has different menu setting patterns because of the different food items they have on offer. In their outlets, different food items are prepared and displayed at different parts of the day. Unprocessed IRFVs displayed their fruits on trays for customers to buy. For both traditional and processed IRFVs, the presentation of food for sale differed throughout the day.

*“…In the morning, as early as possible, I prepare breakfast food (rice, beans, yam and stew) and in the afternoon till evening, I sell mashed foods such as Amala, Eba, Semovita (TFV 14, age 43).”*

*“…I always fry puff puff before any other thing, then followed by egg roll, then buns in the afternoon and lastly fish roll in the evening (PFV 3, age 39).”*

The composition of the menus is similar on a daily basis for most categories of food vending. They stick to their routine menu and make changes only under some special circumstances. This implies that, in particular processed IRFVs find it difficult to expand their regular menu-setting which may constrain improvements in the diversity of food groups provisioned.

#### Changes embedded in informal food vending practices

Nevertheless, over time IRFV practices have changed in terms of their mode of operation, sale, provisioning period, menu-setting, price of raw food materials, and the variety of food groups provisioned. For example, according to this TFV respondent:

*“…I started with mobile food vending, selling only rice and beans and after some years I got a shop and I started selling other ready-to-eat food items such as fufu, amala eba, semovita (TFV 10, age 35).”*

Having a stationary food outlet influences the number of food groups provisioned as it allows vendors to operate from a foundation of stability and routine, in fixed spaces where they can stock, store and prepare food materials, resources and utensils. The price of raw food materials does not really influence the food diversity provisioned rather the quantity dished out to consumers is adjusted regularly based on the changes in price of raw food materials. Some vendors have transformed from a processed to a traditional food vending due to the continuous demand from their customers for particular kinds of foods giving them the opportunity to provide more food groups. Also changes in the timing of food provisioned were observed:

*“…I used to provide food only in the evening before (jollof-rice, moin-moin and smashed foods) but now I provide throughout the day and have added other food items to my menus (TFV 15, age 49).”*

In summary, it is evident from our findings that informal ready-to-eat food vending practices are formed through the integration of an array of practice-elements. These different practice-elements influence the diversity of food groups provisioned and thereby the dietary outcomes. Although there are some dimensions of practice-elements that cut across all the three food vending categories, there are others that are peculiar to a particular food vending category. Differences across food vending types were observed in terms of the diversity of food groups provisioned, their healthiness, and as well as the daily activities underpinning the operations. The study informs on the skills and competences needed and shows that the raw food materials and the variety of food groups provisioned are guiding the meaning of the practice. Informal ready-to-eat food vending is an important component of the food supply system in urban Nigeria and each element of these practices is vital for its overall functioning. Likewise, the study shows the variability in the categories of food vending in terms of the capacity and required resources. Traditional IRFV showed most capabilities and resources required for the provisioning of more diverse food groups. Consequently, we found that the more diversified the food vending practice, and the more complex the skills and competences underpinning it, the higher the vendor’s income. Some vendors have transformed from processed to traditional IRFV vending due to the continuous demand for particular kinds of foods from their customers giving them the opportunity to provide more different and diverse food groups.

## Discussion and conclusion

This paper aimed to gain deeper insights into urban informal ready-to-eat food vending practices in the context of nutritional health and food diversity. Building on and moving beyond existing work (in nutrition and other fields), this sociological analysis focused explicitly on understanding the everyday performances, skills and competences of different food vending practices.

We analyzed how the different components of out-of-home food vending practices connect with the diversity of food groups provisioned. Three key categories were identified as central in the IRFV sector of Ibadan: traditional, processed and unprocessed food vending practices. Across these three categories our analysis revealed broad differences between their practices in terms of nutritional context and diversity of food groups provisioned. These differences in diversity and nutrition were further found to be related to the practice components in terms of skills and competences, materials and resources needed to sustain operations. Such competences and resources include skills required for food procuring and preparation, material resources in terms of ingredients, cooking utensils, space and storage units needed, as well as the temporal rhythm of the practice in terms of hours spent on food preparations and timing of different meals. Overall the analysis revealed that traditional food vending practices are of the highest diversity and nutritional content, with a greater capacity to support healthy and diverse diets among out-of-home poor consumers. Compared to processed IRFV, the daily performance of traditional IRFV practices depends on competences concerning food knowledge and preparation that require more learning which is acquired through more complex socialization processes as well as access to material resources such as fridge’s, storage units, fixed outlets, etc. Understanding the ways in which of IRFV practices differ in terms of the constituent elements comprising them have important implications and directions for policies focused on improving dietary diversity among the urban poor.

Firstly, the three categories of IRFV provide different numbers of food groups. Traditional IRFV, involving more activities and functions, provides a broader range of food groups serving more people than the other two categories. Along the practice-chain of food vending, different sequential stages influence the diversity of food groups provisioned. Understanding these elements can enable opportunities for policy actors to intervene, monitor, and adjust the core practice. Each food vending category needs a particular type of support to deliver more diverse food groups and healthy dietary outcomes. Unprocessed IRFV practices are rather simple and mainly involve procurement and displaying while traditional IRFV practices involve a broad range of supplying, preparing and managerial activities. The changes occurring among the IRFV practices also differed per category. Some traditional food vendors operated first as processed food vendors but due to the evolving demand from their customers they changed the kinds of food provisioned into offering more traditional meals. However, unprocessed food vendors remained unchanged except when they increased the variety of fruits provisioned. The structure of traditional IRFVs, shows the characteristics to enable the provision of diverse diets, while there is also an opportunity for expansion, restructuring and repositioning in processed IRFVs to enable the diversity of provision by readjusting and reorganizing its practice-elements.

Secondly, our findings also revealed that food vending interrelates with other food related provision practices by engaging in transactions to procure the necessary food ingredients. By operating in vendor-vendor relationships they have multiple interactions, which may contribute to more effective and efficient performances, functions and deliveries in food vending. IRFV practices are connected with input suppliers at short and long distances, creating a dynamic bundle of practices. So, if there is urgent need for food ingredients in smaller quantities, the ingredient suppliers at short distance are considered while otherwise open markets at larger distance are patronized. The diversity of the food groups provisioned by the IRFV practices is directly influenced by the ease in accessing raw food materials. We showed that an increase in the availability of a broad variety of food ingredients leads to a greater variety of ready-to-eat food groups provisioned. The close relationship between the food vendors and the different food ingredient suppliers allows them to access food ingredients on credit. Supermarkets and expensive restaurants form an inspiration in shaping and transforming food vending practices, as many food vendors tend to imitate their practices to improve their own.

Thirdly, our findings indicate that traditional food vending seems to be more diversified when compared with processed and unprocessed food vending, although some of the food groups provided are consumed in small quantities only. The number of food groups provided is higher because some of them are consumed in small quantities combined in particular dishes. Unprocessed food vending is mostly limited to two food groups because of what customers expect them to provide. With respect to the diversity of food groups provisioned, processed food vending is positioned in between the two other categories. When analyzing the food groups provisioned as regards to their health score, we found that only seven healthy food groups are provisioned among the three categories of food vending, whereby traditional IRFV offered the largest number of healthy food groups.

In conclusion, the study highlights the contributions from a social practice understanding of IRFV and how important the interactions between the practice-elements are to achieve health and diversity in the food provisioned. This approach also provides the opportunity to identify the critical element(s) across the three categories of IRFV and to identify opportunities for (policy) interventions and applications to improve of IRFV practices and enable diverse diets to be supplied to urban consumers. Improving the success of IRFV can for instance be achieved by improving the necessary skills and competences of vendors to procure diverse raw food materials and to transform these into edible meals. Also, effective stocking and storage facilities are needed to support the provisioning of more diversified food groups. Critical skills and competences are cooking skills, bargaining and purchasing skills and nutritional knowledge. Such practice-element(s) need attention for future intervention to achieve health and diversity in informal ready-to-eat food provisioned as a component of an urban food supply system that is accessible to urban poor. From a practice perspective, recruitment is important and we found that IRFV is attractive for younger members in the family of vendors and that most learning takes place within the household. The shows how IRFV is an everyday routinized practice that is transmissible at both the family and community levels. We also found that the levels of healthy food provided by IRFV practices could be improved. In general, this study showed that a social practice approach generates detailed empirical results on the practice-elements of IRFV which can adequately inform policy making as well as aid smooth policy application and implementation to support more diversity in the food provided through IRFV practices.

## Data Availability

The data for the study is available upon request from the corresponding author.

## Abbreviations

GIS: Geographical Information System
IRFV: Informal Ready-to-eat Food Vending
LGA: Local Government Area
PFV: Processed Food Vendor
SPT: Social Practice Theory
TFV: Traditional Food Vendor
UPFV: Unprocessed Food Vendor
